# Gender specific click and tone burst evoked ABR datasets from mice lacking the Ca_v_3.2 T-type voltage-gated calcium channel

**DOI:** 10.1186/s13104-019-4169-4

**Published:** 2019-03-20

**Authors:** Andreas Lundt, Christina Henseler, Carola Wormuth, Julien Soos, Robin Seidel, Ralf Müller, Muhammad Imran Arshaad, Karl Broich, Jürgen Hescheler, Agapios Sachinidis, Dan Ehninger, Anna Papazoglou, Marco Weiergräber

**Affiliations:** 1 Experimental Neuropsychopharmacology, Federal Institute for Drugs and Medical Devices (Bundesinstitut für Arzneimittel und Medizinprodukte, BfArM), Kurt-Georg-Kiesinger-Allee 3, 53175 Bonn, Germany; 2 Federal Institute for Drugs and Medical Devices (Bundesinstitut für Arzneimittel und Medizinprodukte, BfArM), Kurt-Georg-Kiesinger-Allee 3, 53175 Bonn, Germany; 3 Cognitive Neurophysiology, Department of Psychiatry and Psychotherapy, University Hospital Cologne, University of Cologne, Faculty of Medicine, Kerpener Str. 62, 50937 Cologne, Germany; 4 Institute of Neurophysiology, University of Cologne, Faculty of Medicine, Robert-Koch-Str. 39, 50931 Cologne, Germany; 5 Molecular and Cellular Cognition, German Center for Neurodegenerative Diseases (Deutsches Zentrum für Neurodegenerative Erkrankungen, DZNE), Sigmund-Freud-Str. 27, 53127 Bonn, Germany

**Keywords:** Amplitude, Auditory brainstem responses, Calcium channel, Ca_v_3.2, Click, Monaural, Sound pressure level, Threshold, Tone burst, Transgenic mice, T-Type

## Abstract

**Objectives:**

Voltage-gated Ca^2+^ channels (VGCCs) are of central relevance in regulating Ca^2+^ influx into living cells. The low-voltage activated (LVA) Ca_v_3 T-type Ca^2+^ channels are widely distributed throughout the brain including the peripheral auditory system and ascending auditory tract. Their exact role in auditory information processing is still not fully understood. Within the LVA subgroup, Ca_v_3.2 T-type Ca^2+^ channels seem to be of special importance as qPCR revealed a steady increase in Ca_v_3.2 transcript levels over age, e.g. in the cochlea and spiral ganglion neurons (SGN). Furthermore, pharmacological studies suggested an association between Ca_v_3.2 expression and both age-related and noise-induced hearing loss. Given the potential functional relevance of Ca_v_3.2 VGGCs in sensorineural hearing loss, we recorded gender specific auditory evoked brainstem responses (ABRs) upon both click and tone burst presentation. Here we present auditory brainstem response (ABR) data from Ca_v_3.2^+/+^, Ca_v_3.2^+/−^ and Ca_v_3.2^−/−^ mice from both genders which are of value for researchers who want to evaluate how Ca_v_3.2 loss affects basic auditory parameters, e.g. click and tone burst based hearing thresholds, amplitude growth function and peak latencies.

**Data description:**

Information presented here includes ABR data from age-matched female and male Ca_v_3.2^+/+^, Ca_v_3.2^+/−^ and Ca_v_3.2^−/−^ mice and technical aspects of the auditory recording protocol. Data were recorded using a commercially available ABR setup from Tucker Davis Technologies Inc. (TDT). Raw data files (arf.-file format) were exported as txt.-files with free access for analysis.

## Objective

Voltage-gated Ca^2+^ channels are key players in regulating cellular Ca^2+^ homeostasis. Only few Ca^2+^ channels have been functionally related to auditory information processing including Ca_v_1.3 L-type Ca^2+^ channels, the ablation of which results in congenital deafness. Recent animal studies suggested that Ca_v_3.2 T-type Ca^2+^ channels might play a role in age- and noise-induced hearing loss as ablation of the channel seemed to protect from sensorineural hearing loss. On the other hand, Ca_v_3.2 Ca^2+^ channels were reported to exhibit increased expression with age in the inner ear/spiral ganglion neurons pointing to an important functional role in the auditory system. Interestingly, no auditory profiling of Ca_v_3.2^+/−^ and Ca_v_3.2^−/−^ mice had been performed to unravel the physiological involvement of Ca_v_3.2 in the peripheral and ascending auditor tract. To do so, we carried out click and tone burst evoked auditory brainstem response (ABR) recordings from Ca_v_3.2^+/+^, Ca_v_3.2^+/−^ and Ca_v_3.2^−/−^ mice from both genders. Monaural recording results in all three lines were analyzed for threshold alterations and differences in peak amplitudes and peak latencies and submitted elsewhere. Raw ABR data were exported as txt.-files to provide free access and to enable researchers to carry out their own ABR data analysis, including further investigation of binaural recordings or application of additional manual and/or automatic analytical tools (Table 1).

**Table 1:** Overview of data files/data sets [2]

Label	Name of data file/data set	File types (file extension)	Data repository and identifier (DOI or accession number)
Data description 1	Click header description	.docx file format	Mendeley Data (http://dx.doi.org/10.17632/dms4hsd75s.1)
Data description 2	Tone burst header description	.docx file format	Mendeley Data (http://dx.doi.org/10.17632/dms4hsd75s.1)
Exported data set 1	Female Ca_v_3.2^+/+^ Animal # 1–12 (click and tone burst evoked ABRs)	.txt file format	Mendeley Data (http://dx.doi.org/10.17632/dms4hsd75s.1)
Exported data set 2	Female Ca_v_3.2^+/−^ Animal # 1–8 (click and tone burst evoked ABRs)	.txt file format	Mendeley Data (http://dx.doi.org/10.17632/dms4hsd75s.1)
Exported data set 3	Female Ca_v_3.2^−/−^ Animal # 1–8 (click and tone burst evoked ABRs)	.txt file format	Mendeley Data (http://dx.doi.org/10.17632/dms4hsd75s.1)
Exported data set 4	Male Ca_v_3.2^+/+^ Animal # 1–11 (click and tone burst evoked ABRs)	.txt file format	Mendeley Data (http://dx.doi.org/10.17632/dms4hsd75s.1)
Exported data set 5	Male Ca_v_3.2^+/−^ Animal # 1–7 (click and tone burst evoked ABRs)	.txt file format	Mendeley Data (http://dx.doi.org/10.17632/dms4hsd75s.1)
Exported data set 6	Male Ca_v_3.2^−/−^ Animal # 1–9 (click and ton burst evoked ABRs)	.txt file format	Mendeley Data (http://dx.doi.org/10.17632/dms4hsd75s.1)

## Data description

### Experimental animals

Ca_v_3.2 transgenic mice [1] from Mutant Mouse Resource and Research Centers (MMRRC: 009979-MU; strain name: B6.129-*Cacna1h*^*tm1Kcam*^/Mmmh) were maintained in the C57Bl/6J background. For subsequent ABR recordings, Ca_v_3.2^+/+^ controls, heterozygous Ca_v_3.2^+/−^ and homozygous null mutant Ca_v_3.2^−/−^ mice (55 animals in total) were used from both age-matched genders with the following characteristics: Males: Ca_v_3.2^+/+^: n = 11 (♂), weight 32.82 ± 0.58 g; Ca_v_3.2^+/−^: n = 7 (♂), weight 33.11 ± 0.81 g; Ca_v_3.2^−/−^: n = 9 (♂), weight 29.09 ± 0.75 g. Females: Ca_v_3.2^+/+^: n = 12 (♀), weight 24.09 ± 0.41 g; Ca_v_3.2^+/−^: n = 8 (♀), weight 23.50 ± 0.41 g; Ca_v_3.2^−/−^: n = 8 (♀), weight 22.10 ± 0.43 g.

### ABR recording procedure

For recording of monaural bioelectrical auditory potentials, subdermal stainless steel electrodes were inserted at the vertex, axial the pinnae [(+) electrode] and ventrolateral of the right pinna [(−) electrode]. The ground electrode was positioned at the hip of the animal. To verify proper electrode positioning/conductivity, impedance measurements of all electrodes (< 5 kΩ) were carried out prior to each recording [Lundt A, Seidel, Robin, Soos J, Henseler C, Müller R, Bakki M, Arshaad IM, Ehninger D, Hescheler J, Sachinidis A, Broich K, Wormuth C, Papazoglou A, Weiergräber M. Ca_v_3.2 T-type calcium channels are physiologically mandatory for the auditory system despite their devastating role in sensorineural hearing loss. Neuroscience, unpublished].

All ABR recordings were performed under free field conditions using a single loudspeaker (MF1 Multi-Function Speaker, TDT, USA) which was positioned 10 cm opposite to the rostrum of the animals.

The SigGenRZ software (TDT) was used to program stimulus protocols for click and tone bursts. The bioelectrical ABR signals recorded from the subdermal electrodes were transferred to a head stage (RA4LI, TDT) and forwarded to the preamplifier (RA4PA, TDT) with 20-fold amplification.

ABR data acquisition was carried out at a sampling rate of 24.4 kHz and signals were bandpass filtered (high pass 300 Hz, low pass 5 kHz) using a 6-pole Butterworth filter. The individual ABR data acquisition time was 25 ms starting with a 5 ms baseline period prior to the individual acoustic stimulus onset (pre ABR baseline) and exceeding the 10 ms ABR section by another 10 ms baseline (post ABR baseline) [Lundt et al., unpublished].

Two types of acoustic stimuli were applied for ABR recordings using the SigGenRZ software (TDT) and applied via the TDT BioSigRZ platform. The first stimulus entity was a click of 100 µs duration, with alternating polarity (switching between condensation and rarefaction).

The second stimulus entity was a 4.5 ms tone burst (transient sinusoidal plus) of alternating polarity with Hann envelope rise and fall times of 1.5 ms duration. The frequency range covers 1–42 kHz in 6 kHz steps. All acoustic stimuli were applied 300 times at a rate of 20 Hz for averaging.

Sound pressure levels (SPL) were increased in 5 dB steps for clicks and 10 dB steps for tone bursts, starting from 0 dB up to 90 dB (increasing SPL mode). Sound pressure levels for tone bursts within the range of 1–42 kHz were calibrated each day prior to recording [Lundt et al., unpublished].

## Limitations

ABR data presented here were performed under standard free field conditions. Data were recorded from age-matched animals of ~ 20 weeks. We did not record from animals of different age.

## Abbreviations

ABR: auditory brainstem response
SPL: sound pressure level
